# Glycerol Kinase 2 as a Metabolic Sentinel for Human Sperm Motility and Male Fertility

**DOI:** 10.3390/biom15091249

**Published:** 2025-08-29

**Authors:** João S. Oliveira, Rúben J. Moreira, Ana D. Martins, Marco G. Alves, Pedro F. Oliveira

**Affiliations:** 1 LAQV-REQUIMTE and Department of Chemistry, University of Aveiro, 3810-193 Aveiro, Portugal; joaooliveira23@ua.pt (J.S.O.); rubenjesusmoreira@ua.pt (R.J.M.); anacdmartins@gmail.com (A.D.M.); 2 iBiMED-Institute of Biomedicine, Department of Medical Sciences, University of Aveiro, 3810-193 Aveiro, Portugal; marcoalves@ua.pt

**Keywords:** male infertility, assisted reproductive technologies, spermatozoon, asthenozoospermia, sperm motility, glycerol, glycerol kinase 2

## Abstract

Male infertility affects 8–12% of couples worldwide and is solely responsible in up to 30% of cases. Assisted Reproductive Technologies (ARTs) provide potential solutions, particularly in conditions where spermatozoa display structural abnormalities or impaired motility, such as asthenozoospermia. Sperm metabolism demonstrates remarkable flexibility, shifting between glycolysis and oxidative phosphorylation to produce ATP required for motility. Glycerol kinase 2 (GK2) phosphorylates glycerol in the sperm midpiece, generating glycerol-3-phosphate, a key intermediate in glycolysis, lipid metabolism, and oxidative phosphorylation. The localization of GK2 suggests not only a regulatory role in sperm metabolism but also a possible association with VDAC proteins, contributing to ADP-ATP exchange between the cytosol and mitochondria. Elucidating the role of GK2 in spermatozoa is of particular relevance, as this enzyme not only contributes to key metabolic pathways but may also interact with VDAC proteins, influencing mitochondrial function and energy exchange. Such interactions could play a pivotal role in regulating sperm motility. A deeper understanding of these mechanisms could position GK2 as a valuable biomarker: in scenarios where GK2–VDAC interactions are confirmed, it may guide optimized sperm selection methods in ARTs, whereas the absence or impairment of such interactions could serve as a diagnostic indicator in asthenozoospermic men.

## 1. Introduction

Male fertility is a complex interplay of physiological events crucial for successful impregnation, including spermatogenesis, hormonal regulation, testosterone production, and sperm functionality. Understanding and optimizing these components is essential for assessing and enhancing male fertility [1]. In recent years, there has been a notable surge in the prevalence of male infertility, contributing to a growing global concern [2]. Infertility, which refers to the inability to conceive after one year of regular unprotected intercourse [3], affects approximately 8 to 12% of couples worldwide, with male infertility independently representing 20 to 30% of cases and 50% of all infertility cases [2]. As of 2019, an estimated 56 million men grapple with fertility issues. This marks a substantial increase of 76.9% compared to the figures recorded in 1990, a period during which the global population rose only by 46% [2]. Infertility assessment involves a comprehensive examination of clinical history, including health conditions, infertility duration, and family history [1,3,4]. Genetic testing makes possible the diagnosis of genetic disorders causing infertility [4]. Physical examination assesses sexual development and genitalia, revealing conditions like hypogonadism, varicocele, and testicular abnormalities [5]. Laboratory tests include hormone profiling, which measures follicle-stimulating hormone (FSH), luteinizing hormone (LH), and total testosterone levels, while semen analysis detects abnormalities in spermatozoa [5]. A detailed evaluation is essential for diagnosing and addressing male infertility.

Advanced interventions have been developed to address the diverse causes of male infertility. Assisted Reproductive Technologies (ARTs) are medical procedures to aid an individual or couple who grapple with fertility issues. ARTs include procedures such as *in vitro* fertilization (IVF) and intracytoplasmic sperm injection (ICSI). In IVF, fertilization is performed by placing the gametes in contact in an artificial environment and the embryo formed is transferred to the uterus. In ICSI, a single spermatozoon is directly injected into the cytoplasm of the oocyte and the embryo formed is also transferred to the uterus [6]. Both of these procedures are particularly helpful in cases of low sperm motility [7].

Loss of sperm motility is one of the leading causes of male infertility, a condition known as asthenozoospermia. The World Health Organization (WHO) uses a 5th percentile, with a 95% confidence interval, to define reference values for semen parameters, including sperm motility. For total motility (progressive and non-progressive), the lower reference limit is 42%. For progressive motility, the lower reference limit is 30% [8]. Men diagnosed with asthenozoospermia display sperm with impaired motility, falling below these reference values, and often present a reduced sperm count. Asthenozoospermia may occur as an isolated disorder or in conjunction with other sperm abnormalities, such as teratozoospermia (abnormal sperm morphology) and oligozoospermia (low sperm count), a condition referred to as oligoasthenoteratozoospermia [8]. Asthenozoospermia is commonly associated with ultrastructural defects in spermatozoa and the presence of antisperm antibodies, varicocele, and urogenital infections. Additionally, lifestyle factors can also contribute to the development of this condition [9,10].

Semen analysis remains the gold standard for the clinical evaluation of male infertility, especially for asthenozoospermia. However, its diagnostic utility is limited, as it primarily identifies abnormalities in motility parameters without providing comprehensive information regarding the underlying etiologies of this anomaly and overall infertility [11]. Indeed, the diagnostic utility of standard semen analysis is limited as it often fails to detect crucial molecular alterations at the chromatin level. The integrity of sperm chromatin is vital for protecting the paternal genome, with nuclear proteins like protamines being essential for its stability and compaction. Recent evidence has demonstrated that environmental pollutants can alter the properties of these sperm nuclear basic proteins, inducing damage that is not visible with a conventional spermiogram [12]. This diagnostic gap means that underlying molecular causes of infertility may be missed, reinforcing the critical need to develop novel diagnostic tools, particularly at the molecular level, to better understand the causes of male infertility. In this context, molecular biomarkers represent a promising approach, offering the potential to enhance diagnostic accuracy and elucidate underlying pathophysiological mechanisms. Glycerol kinase 2 (GK2), a testis-specific gene involved in mitochondrial function and sperm motility [13], has emerged as a potential biomarker, with alterations in its expression being associated with impaired spermatogenesis, motility, and, consequently, male infertility.

GK2 is the isoform of the enzyme glycerol kinase (GK) that is expressed in the testis and fetal tissue, contrary to glycerol kinase 1 (GK1), and is mainly active in non-testicular somatic cells [14]. These isoforms of GK catalyze the phosphorylation of glycerol into glycerol-3-phosphate (G3P), a key intermediary in various metabolic pathways, including glycolysis, gluconeogenesis, and glyceroneogenesis [13]. Although glycerol is predominantly metabolized in the liver [15], the presence of GK2 in the testis highlights the significance of glycerol and G3P in testicular metabolism [13].

Although GK2 has been implicated in several processes in spermatozoa of mammalian species, including mice and boar, evidence regarding its specific role in human spermatozoa is still lacking, which makes it difficult to fully understand its metabolic significance in humans. Therefore, this review aims to provide a comprehensive understanding of GK2’s role in human spermatozoa. We will explore its participation in intermediary metabolism, focusing on its involvement in key metabolic pathways vital for sperm motility and evaluating its functional importance in maintaining sperm function, particularly its connection to mitochondrial activity and energy regulation. Unveiling the functional importance of this enzyme on spermatozoa may disclose new paths for the diagnosis and treatment of male infertility, providing a crucial tool as a biomarker of motility-related disorders (e.g., asthenozoospermia) or serving as a powerful sperm selection method for ARTs.

## 2. The Landscape of Assisted Reproductive Technologies (ARTs)

According to the American Centers for Disease Control (CDC), ARTs do not include procedures where only sperm is handled, such as intrauterine injection (IUI), and procedures where oocytes are stimulated without the intention of retrieving them. At present, the available and most used ARTsemployed for the management of male infertility are IVF and ICSI [16]. IVF is a procedure where fertilization is performed in an artificial environment, where the embryo is transferred to the uterus. IVF is recommended for male patients who suffer from low sperm counts and/or motility (but enough to allow fertilization in the laboratory), infertility related to sperm antibodies, and idiopathic infertility. On the other hand, ICSI is indicated for men suffering from severe male infertility, caused by anomalies in the sperm (oligozoospermia, asthenozoospermia, teratozoospermia, and azoospermia), as well as ejaculatory problems [7].

The process of IVF starts with the retrieval of the oocyte by transvaginal recovery, after undergoing an optimal stimulation protocol. The healthiest and most motile sperm are selected and placed in a culture dish with medium for the insemination of the oocytes, followed by incubation under ideal pH and temperature conditions. Fertilization assessment is made 16 to 20 h after the insemination, and fertilized oocytes are incubated for 24 h in fresh embryo culture media. The ideal number of embryos transferred to the patient is decided by the specialist responsible for the procedure and normally does not exceed three [6]. This procedure implies the random choice of gametes; however, this randomness may not guarantee the selection of the most competent spermatozoa, potentially limiting the efficiency of fertilization and embryo development. Therefore, integrating more precise sperm selection strategies, based on molecular or functional markers, could enhance the success rates and outcomes of ART procedures.

Besides the aforementioned constraint, ARTs have several limitations, including the association with a slightly increased risk of post-natal complications, such as anatomic birth defects [17], risk of developing disorders in the autism spectrum (caused by exposure to progesterone) [18], and rare syndromes, such as Beckwith–Wiedemann, Prader–Willi, and Silver–Russell syndromes [19]. Additionally, building ART centers and the treatment itself are costly and not equally accessible in all countries, as the financial burden of an ART treatment differs in different countries, as a result of the absence of financing from the government or the yearly income of the patients [20]. ARTs also involve ethical and religious issues: Islamism and Hinduism only accept the birth of a child through ARTs if the gametes come from a married couple (a third party cannot be involved), whereas the major branches of Christianity do not accept any form of ART [21].

Nonetheless, ARTs provide a unique opportunity for couples unable to conceive. Several benefits are associated with the use of these technologies beyond overcoming infertility—preservation of fertility, genetic testing, and an option for same-sex couples or single parents. ARTs provide an opportunity for fertility preservation, particularly for patients undergoing treatment for conditions that may affect fertility, such as cancer, HIV, nononcologic conditions (for example, autoimmune diseases), or for patients susceptible to fertility rate decline [22]. Fertility preservation includes cryopreservation and surgical retrieval of oocytes and sperm, oocyte vitrification, or gamete donation, among other techniques under development (artificial ovaries, uterus transplantation, or testicular organoids) [23]. The collected and preserved gametes can be utilized at the desired timing through ARTs. Preimplantation genetic testing (PGT) is used for screening embryos for genetic conditions before uterine implantation. It improves pregnancy success rates and reduces the risk of genetic disorders in offspring. PGT is recommended in situations of several previous pregnancy losses or implantation failures, advanced maternal age, family history of genetic conditions, and male-related infertility [24,25]. ARTs also offer the possibility for the birth of genetically related children for same-sex couples or single parents. For both cases, these technologies involve a complex process for male patients, who require an oocyte donor as well as a gestational carrier, in contrast to equal situations involving female patients, who need only a sperm donor. Nonetheless, understanding the needs of these growing minorities will result in an increased use of ARTs from these groups, providing a wider range of options other than adoption or foster, and offering the best treatment given the ideas of family planning each group has [26].

## 3. Spermatozoa: Structure, Function, and Motility

Spermatozoa are male gametes that carry the genetic material and that, when fully matured, are able to successfully fertilize the oocyte. Spermatogenesis is the biological process responsible for the formation of spermatozoa. It involves the differentiation of an undifferentiated diploid cell, known as a spermatogonium, into a highly specialized haploid cell, the spermatozoon [27]. This complex process encompasses a series of mitotic and meiotic divisions, along with extensive cellular remodeling. Spermatogenesis can be divided into three distinct phases: proliferation and differentiation of spermatogonia, meiosis, and spermiogenesis [28]. All of these phases occur within the seminiferous tubules, a network of highly convoluted tubules, located within the testes. These tubules are separated by interstitial compartments containing Leydig cells, blood vessels, lymphatic structures, and nerves. The seminiferous tubules are structurally organized into two distinct compartments: the basal compartment, corresponding to the basement membrane, and the adluminal compartment, representing the lumen, divided by intercellular tight junctions [27]. Surrounding the tubules is a three-layered peritubular tissue structure, consisting of an outer adventitial layer composed of fibrocytes, a middle layer of myoid cells, and an inner peritubular layer primarily composed of collagen [29]. The seminiferous tubules are lined with Sertoli cells, which extend from the basement membrane to the lumen. These somatic cells serve essential functions in spermatogenesis, including the nourishment of developing germ cells and the phagocytosis of apoptotic germ cells and residual bodies [27].

Spermatogenesis begins with spermatogonia, which undergo a series of mitotic divisions to both maintain the stem cell pool and produce differentiated cells that give rise to primary spermatocytes [30]. Primary spermatocytes undergo the first meiotic division in the basal compartment of the seminiferous tubules. During this division, each primary spermatocyte gives rise to two secondary spermatocytes, which subsequently undergo the second meiotic division in the adluminal compartment, producing four haploid spermatids carrying either (22,X) or (22,Y) chromosomes [27]. These spermatids then undergo spermiogenesis, which encompasses a series of morphological changes happening in six different stages. During these stages, spermatids undergo numerous morphological changes, including the development of mitochondria and the Golgi complex; the development of the acrosomal vesicle, proximal centriole, and axial filament; completion of the acrosome formation; formation of the intermediate piece and tail; and progressive condensation of the nucleus [31]. Upon completion of these stages, a functional spermatozoon with fully compacted chromatin is formed.

Upon completion of spermatogenesis, spermatozoa are morphologically developed but remain immature and lack motility. To acquire the capacity to successfully fertilize an oocyte, spermatozoa must undergo a series of modifications, including epididymal maturation, capacitation, and the acrosome reaction. The journey through the epididymis represents a critical phase in this process, as it facilitates the acquisition of both primary and activated motility [32]. This stage of sperm maturation will be explored in detail in the subsequent sections of this review.

### 3.1. Structural Basis of Sperm Function

**Figure 1:**
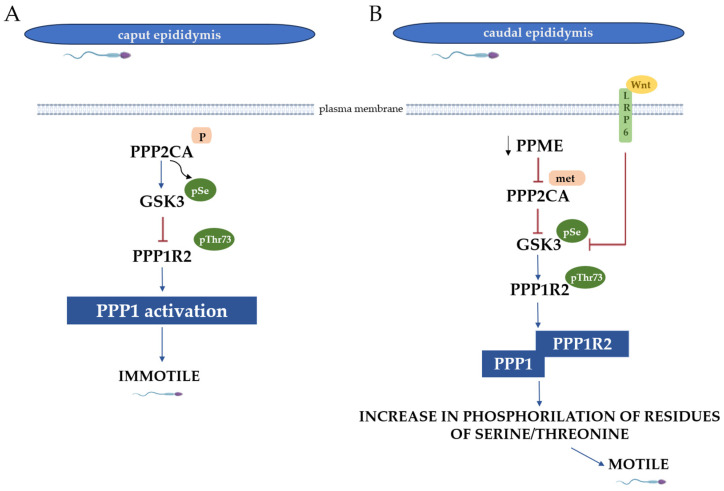
Diagrammatic representation of the signaling events in the caput and caudal sperm during the acquisition of motility in the epididymis. (**A**) In the caput sperm, PPP2CA is activated due to phosphorylation, which causes a dephosphorylation at serine residues in GSK3. This process makes GSK3 active, triggering a phosphorylation in threonine 73 of PPP1R2, rendering it inactive. The inactivation of PPP1R2 prohibits its binding to PPP1 and the following activation, causing a dephosphorylation of serine and threonine residues, resulting in immotile spermatozoa. (**B**) In caudal sperm, a decrease in PME1 activity causes an increase in PPP2CA methylation, inhibiting it. Phosphorylation in serine residues of GSK3 and the binding of Wnt proteins to LRP6 promote the inhibition of GSK3. This inhibition leads to decreased threonine 73 phosphorylation in PPP1R2, allowing its binding to PPP1. Consequently, PPP1 activity is inhibited, allowing an increase in phosphorylated serine and threonine residues, resulting in motile spermatozoa. Legend: GSK3—glycogen synthase kinase 3; PPP2CA—phosphoprotein phosphatase 2A isoform alpha; PPME1—protein phosphatase methylesterase 1; PPP1—phosphoprotein phosphatase 1; PPP1R2—PPP1 regulatory subunit 2; and LRP6—lipoprotein receptor-related protein 6 receptor.

The structure of a spermatozoon comprises three primary components: the head, the neck/midpiece region, and the tail/flagellum (Figure 1). The oval head possesses a nuclear envelope—with condensed chromatin in which the histones have been largely replaced by protamines for higher stability, compaction, and protection against damage [33]—and a well-defined acrosome occupying 40 to 70% of the anterior region. The midpiece region serves as the structural nexus binding the flagellum to the head of the sperm. In human sperm, the midpiece is characterized by the presence of 12 or 13 gyres of mitochondria, each containing two mitochondria *per* gyre, with a comparatively lower mitochondrial count often associated with individuals with asthenozoospermia [34]. The tail is further divided into three main components—principal piece, mid-piece, and end piece. It contains the axoneme—a structure containing nine outer microtubules and one central doublet (a total of eleven microtubules), connected by radial spokes and dynein arms. The axoneme surrounds the entirety of the tail, along with periaxonemal structures (a helix formed by 10–12 mitochondrial sheets), which cover everything but the end piece [35]. The principal piece is covered by a structure with two longitudinal columns linked with circumferential ribs, called fibrous sheets (FSs), acting as a framework for proteins involved in metabolic pathways in sperm, such as glycolysis and cAMP-dependent signaling transduction and support [36].

### 3.2. Molecular Events for Spermatozoa Motility

Spermatogenesis produces morphologically developed sperm cells; however, these cells remain functionally immature and lack motility upon their release from the testis. The acquisition of motility is a critical aspect of sperm maturation, enabling them to navigate the female reproductive tract in pursuit of the oocyte. As spermatozoa progress through the epididymis, they undergo significant biochemical and structural modifications, particularly in the composition of their plasma membrane. These changes facilitate the development of distinct motility patterns, ultimately culminating in hyperactivated motility [32].

Various signaling pathways are involved in the acquisition of motility in spermatozoa. As sperm travel through the epididymis—divided into the caput (adjacent to the testicles), corpus, and cauda (adjacent to the vas deferens) [37]—a key signaling event regulating sperm motility occurs. This involves the modulation of phosphoprotein phosphatase 1 (PPP1) activity, which is closely linked to sperm motility. PPP1 regulatory inhibitors (PPP1Rs) have been identified in the head and tail (principal and mid-piece) of human sperm, namely PPP1R subunit 2 (PPP1R2) and PPP1R2 pseudogene 3 (PPP1R2P3) [38].

In the caput sperm, high PPP1 activity renders the sperm immotile, serving as a “brake” system when activated motility is not required. This occurs because of a demethylated and phosphorylated isoform of phosphoprotein phosphatase 2A (PP2A), known as phosphoprotein phosphatase 2A isoform alpha (PPP2CA), which dephosphorylates glycogen synthase kinase 3 (GSK3) at specific serine residues, activating GSK3. The active GSK3 then phosphorylates threonine 73 of PPP1R2, inactivating this inhibitor and, in turn, activating PPP1—resulting in immotile spermatozoa [39].

**Figure 2:**
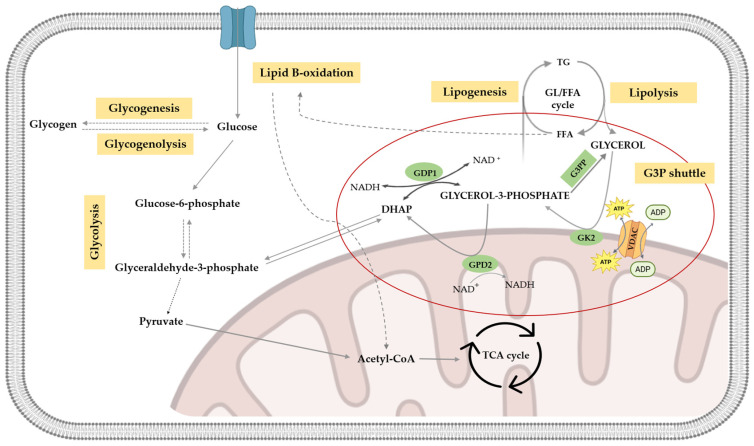
Schematic representation of the reactions occurring in the glycerol metabolism. In the glycerol-3-phosphate (G3P) shuttle, glycerol is transformed into G3P, a reaction catalyzed by GK2, which seems to interact with VDAC proteins. G3P originates from dihydroxyacetone phosphate (DHAP) by action of two isoforms of the GPD enzyme (GPD1, the cytosolic isoform, and GPD2, present in the mitochondria). DHAP can be utilized in metabolic pathways, such as glycogenolysis, glycogenesis, and glycolysis. The latter renders pyruvate, which is transformed into acetyl-CoA and used in the TCA cycle. G3P can enter the GL/FFA cycle, giving rise to the production of glycerol by lipolysis. Legend: DHAP—dihydroxyacetone phosphate; GPD—glyceraldehyde-3-phosphate dehydrogenase; GK 2—glycerol kinase 2; GL/FFAs—glycerides/free fatty acids; FFAs—free fatty acids; TGs—triglycerides; TCAs—tricarboxylic acids; and VDAC—voltage-dependent anion channel.

Conversely, in caudal sperm, PPP1 activity must be maintained low, allowing the acquisition of sperm motility [39]. Phosphorylation of PPP1R2 is mediated by GSK3, whose activity is regulated by Wnt signaling proteins secreted by epididymal principal cells within epididysosomes. These proteins activate the lipoprotein receptor-related protein 6 (LRP6), leading to a cascade that reduces GSK3 inhibition [40]. Consequently, there is a decrease in PPP1R2 phosphorylation at threonine 73, thereby resulting in the activation of this inhibitor. Therefore, this allows the binding to PPP1, resulting in its activity remaining suppressed and, ultimately, in the acquisition of sperm motility [41]. Additionally, GSK3 activity is influenced by PP2A. A decrease in protein phosphatase methylesterase 1 (PPME1) activity increases PPP2CA methylation, rendering PPP2CA inactive. This leads to heightened phosphorylation of GSK3 at serine residues, deactivating GSK3. As a result, PPP1R2 remains active, inhibiting PPP1 and promoting sperm motility [42]. A schematic representation of the molecular events leading to the acquisition of motility by spermatozoa in the epididymis is displayed in Figure 2.

### 3.3. Spermatozoa Abnormalities

Spermatozoa may manifest structural defects across the components of their anatomical organization, affecting the proper functioning of these cells and, therefore, the ability to successfully fertilize. Besides these structural malformations, spermatozoa may also present abnormalities in their motility, count, or viability [43].

These abnormalities can be categorized into two distinct classes. The first class, denoted by the suffix “spermia”, refers to irregularities observed in the ejaculate and encompasses conditions such as aspermia (total absence of ejaculate) and hypospermia (abnormal volume of ejaculate). The second one, denoted by the suffix “zoospermia”, encompasses defects in sperm, including conditions such as azoospermia (absence of sperm cells in the ejaculate), oligozoospermia (low sperm count), teratozoospermia (abnormal structure in a high percentage of sperm cells), necrozoospermia (abnormally low percentage of live sperm cells), and asthenozoospermia (low sperm motility).

Spermatozoa exhibit five distinct types of motility, ranging from complete immobility to rapid, linear movement. This classification includes (1) non-motile spermatozoa, which show no movement; (2) spermatozoa with slow movement but lacking progressive motion; (3) spermatozoa displaying slow, meandering forward progression; (4) spermatozoa moving in a straight line at a moderate speed; and (5) spermatozoa exhibiting high-speed, linear motility [9]. Various factors can contribute to the loss of motility, leading to the prevalence of the first three motility types. One of the primary causes of defective sperm motility is the presence of ultrastructural abnormalities in spermatozoa, particularly in the tail region, which is essential for proper propulsion [44]. However, motility loss can also result from various physiological and environmental factors. For instance, an abnormal enlargement of the testicular veins, known as varicocele, can lead to increased scrotal temperature and possibly oxidative stress, negatively affecting sperm function [45]. Additionally, urogenital infections can induce an inflammatory response, further compromising motility [46]. The presence of antisperm antibodies may also interfere with sperm movement [47]. Excessive reactive oxygen species (ROS) levels can also be detrimental, as they trigger lipid peroxidation, leading to membrane damage and a subsequent decline in motility [48]. Beyond causing membrane damage, excessive ROS can also induce oxidative DNA damage, compromising the genetic integrity of the spermatozoon. The very proteins responsible for organizing and shielding the paternal DNA, such as protamines, can be involved in this process. While these proteins are crucial for chromatin compaction and protection, oxidative stress can alter their structure or function. When altered, their protective capacity can be compromised, paradoxically contributing to oxidative DNA damage and fragmentation. This can negatively impact fertilization and subsequent embryo development [49]. Indeed, exposure to environmental pollution is increasingly recognized as a significant contributor to male infertility. Pollutants such as heavy metals, pesticides, and endocrine-disrupting chemicals can induce oxidative stress and disrupt hormonal regulation, leading to impaired sperm function and motility [50]. Lastly, lifestyle choices can worsen these issues, further impairing sperm motility and overall reproductive potential [10,51].

The integrity of spermatozoa, both structurally and functionally, is vital for successful fertilization. Among the many abnormalities spermatozoa may present, motility disorders are particularly significant, as they directly impact the sperm’s ability to reach and fertilize the egg. These motility impairments may result from intrinsic defects in sperm architecture, especially in the tail, or be influenced by extrinsic factors such as varicocele, infections, immune responses, oxidative stress, and unhealthy lifestyle habits. A comprehensive understanding of these contributing factors is essential for guiding clinical interventions and improving reproductive outcomes in affected individuals.

## 4. Metabolic Pathways in Sperm Function

Fertilization is a dynamic process characterized by the competitive journey of spermatozoa to reach and penetrate the oocyte. Central to the success of this intricate process is the indispensable requirement for sperm motility. The process of motility in sperm is highly energy-dependent, and adenosine triphosphate (ATP) plays a crucial role in facilitating this movement. ATP serves as the primary energy currency in cells, and in the context of sperm motility, it is essential for supporting the coordinated movement of the axoneme (the central structural component of the flagellum) and the surrounding flagellar structures [52].

### 4.1. Metabolic Adaptations for Sperm Propulsion

The principal origin of ATP in sperm remains a subject of ongoing discourse, given the potential involvement of diverse metabolic pathways in their generation [53]. One such pathway is glycolysis, a biochemical process where pyruvate is synthesized utilizing glucose as a substrate. Occurring within the principal piece of the sperm, glycolysis generates energy in the form of ATP and NADH, yielding two ATP molecules per glycolytic cycle. Impaired glycolysis or the absence of glucose may consequently culminate in diminished sperm motility [54,55,56]. ATP can additionally be generated through oxidative phosphorylation, a pathway that takes place within the mitochondria within the midpiece of the sperm. Oxidative phosphorylation is the process by which electrons transported by NADH and FADH_2_ are transferred through the mitochondrial electron transport chain (ETC), culminating in the reduction of oxygen to water. This electron flow drives the generation of a proton gradient across the inner mitochondrial membrane, which is then used by ATP synthase to synthesize ATP from ADP and inorganic phosphate (Pi). This process relies on a sequence of oxidation–reduction reactions, yielding, on average, a total of 30 ATP molecules per glucose molecule consumed. Research findings have suggested a correlation between increased mitochondrial activity and increased sperm motility [57,58]. Conversely, it has been demonstrated that inhibitors targeting the ETC and ATP synthases can induce a reduction in sperm motility [54]. However, there is still debate regarding the extent to which oxidative phosphorylation contributes to ATP production and, consequently, to sperm motility. This arises from the requirement for ATP to traverse the entire length of the flagellum from the midpiece, with potential limitations in supply through diffusion and carrier systems [59]. Notwithstanding the recognized prominence of glycolysis in ATP generation during sperm maturation, the dismissal of oxidative phosphorylation is premature, as both pathways make essential contributions to sperm function. Furthermore, it is plausible that spermatozoa adopt a versatile approach to energy production, potentially employing a combination of glycolysis and oxidative phosphorylation, thereby adapting to the environmental conditions and available substrates [53].

Spermatozoa exhibit remarkable versatility in utilizing diverse substrates for energy metabolism. While glucose stands as the primary substrate, actively participating in various metabolic pathways, it gives rise to various metabolites. This compound plays a crucial role by serving as an intermediate in pyruvate formation, a key component in several metabolic pathways, particularly the tricarboxylic acid (TCA) cycle. In the mitochondria, pyruvate is oxidized and decarboxylated, producing acetyl-CoA, which enters the TCA cycle by combining with oxaloacetate. Throughout the cycle, the oxidation of metabolic intermediates leads to the production of NADH and FADH_2_. These cofactors donate electrons to the ETC, driving the generation of a proton gradient that powers ATP synthesis [60]. Moreover, an array of enzymes involved in lipid metabolism emphasizes the potential use of lipid β-oxidation within the metabolic processes of sperm. This process relies on a sequence of reactions culminating in the transformation of fatty acids into acyl-CoA, transferred by the carnitine shuttle to the mitochondrial matrix, where it undergoes β-oxidation, resulting in the production of acetyl-CoA, NADH, and FADH_2_ [61]. Insights from proteomic studies propose that β-oxidation might play a pivotal role in ATP generation and sperm motility [62], offering a framework for the sustained motility of sperm in instances of substrate deficiency [63]. Additionally, glycogen can serve as a source for glucose production through glycogenolysis under conditions of low substrate availability. However, the accumulation of this metabolite may exert adverse effects that ultimately lead to apoptosis of spermatogenic cells [64]. Nevertheless, sperm possess the ability to exploit alternative substrates, such as lactate (for acrosome reaction) and pyruvate (for ATP production) [65]. The array of available substrates and the inherent flexibility of metabolic pathways underscore the plasticity of sperm metabolism. This metabolic adaptability allows sperm cells to efficiently modulate their energy production processes, conferring a survival advantage under conditions of substrate limitation or environmental stress.

### 4.2. Metabolic Significance of Glycerol in Spermatozoa

Glycerol assumes a pivotal role in intermediary metabolism, potentially serving as a substrate for various metabolic pathways, including glycolysis, lipogenesis, and others [66,67,68,69], with the liver being the central organ of glycerol metabolism [15]. However, spermatozoa also have all the necessary machinery to use glycerol as a metabolic substrate [70,71]. For glycerol to participate in the various metabolic pathways, the addition of a phosphate group is a prerequisite, a reaction catalyzed by the enzyme GK. This enzymatic process produces G3P, which participates as an intermediate in the aforementioned metabolic pathways [72,73]. This reaction is part of the G3P shuttle (as represented in Figure 2), and it emerges as a crucial nexus in the interplay among the glycerol-involved pathways. Within this shuttle, G3P undergoes conversion to dihydroxyacetone phosphate (DHAP) through a reversible reaction catalyzed by glycerol-3-phosphate dehydrogenase—GPD1 (cytosolic isoform) or GPD2 (mitochondrial isoform)—in which NADH/NAD^+^ or FADH_2_/FAD play the role of electron donor or acceptor, respectively. Notably, while GPD1 catalyzes the interconversion of G3P to DHAP, GPD2 is incapable of catalyzing the reverse reaction (DHAP to G3P) [74,75,76]. This enzymatic shuttle affords diverse possibilities in intermediary metabolism, encompassing the generation of DHAP used for glycolysis [77,78], G3P for lipid metabolism [66,67,79], and the facilitation of electron transfer for ATP production through oxidative phosphorylation [80,81,82]. Remarkably, glycerol metabolism also serves as a conduit for supplying intermediaries for both lipogenesis, gluconeogenesis, and the pentose phosphate pathway [83].

In conclusion, glycerol metabolism plays a crucial role in intermediary metabolism, integrating multiple pathways such as glycolysis, lipogenesis, and oxidative phosphorylation. The phosphorylation of glycerol by GK initiates its metabolic utilization, forming G3P, which serves as a key intermediary. The G3P shuttle further regulates the interconversion between G3P and DHAP, facilitating the shift between energy production and biosynthetic processes. Beyond its role in energy metabolism, glycerol metabolism also provides essential intermediates for gluconeogenesis and the PPP, highlighting its metabolic versatility.

## 5. Glycerol Kinases and Male Fertility

Glycerol remains one of the most important intermediates involved in spermatozoa metabolism, serving not only as a potential energy substrate but also playing a central part in several key pathways for motility and maturation. GK2, a testis-specific isoform of the GK family, is essential for the incorporation of glycerol in the spermatozoa metabolism, which is responsible for the phosphorylation of glycerol to G3P, and also plays a role in the formation of the mitochondrial sheath during spermatogenesis. The interactions of this enzyme with several proteins involved in mitochondrial organization suggest that GK2 not only supports energy production but also is responsible for a part of sperm integrity and maturation. This section explores the role of GK2 in sperm biology, exploring the influence of this enzyme on sperm motility and, ultimately, male fertility (see also Table 1).

### 5.1. Role of Glycerol Kinase 2 on Spermatozoa (and Spermatogenesis) and Its Influence on Male Fertility

The role of glycerol in spermatozoa is extensively described. As stated earlier, glycerol plays an important role in several metabolic pathways, serving as an energetic substrate for vital processes, including motility. Moreover, a study from Ribeiro and colleagues showed that, in epididymal sperm maturation, aquaglyceroporin-7 (AQP7) is responsible for glycerol permeability. In this study, AQP7 expression levels correlated with sperm motility, with an increase in AQP7 expression happening simultaneously with the development of sperm hypermotility. This correlation is also proved by the fact that AQP7 is downregulated in asthenozoospermic individuals, resulting in an impaired glycerol permeability mechanism in this low-motility group [84]. Nonetheless, glycerol’s influence on spermatozoa extends beyond its role in metabolic events. Sertoli cells are responsible for the maintenance and regulation of the blood–testis barrier (BTB). These cells use pyruvate from glycolysis (where glycerol can enter as a substrate) for lactate production, meeting energy demands for spermatogenesis and germ cells [13]. Nevertheless, high glycerol concentrations can adversely affect the BTB, causing the death of germ cells and consequently permanent oligozoospermia and azoospermia [13]. Ribeiro and colleagues [85] confirmed the relationship between Sertoli cells and glycerol permeability, demonstrating that cystic fibrosis transmembrane conductance regulator (CFTR), a vital anion channel for fluid homeodynamics in the male reproductive tract, modulates aquaglyceroporin glycerol permeability, which is crucial for avoiding glycerol accumulation and consequent BTB malfunctions.

GK plays a vital role in the male reproductive tract, as it is responsible for the incorporation of glycerol in metabolism through its phosphorylation, yielding G3P. The GK gene family traces its origin to chromosome X, and owing to retrotransposition events, a duplicate copy is retained on chromosome 4. This genomic locus houses the two functional isoforms of GK [14].

The enzyme GK2 is localized within the acrosome and midpiece regions of human spermatozoa [86], where it seems to be bound with the mitochondria [14]. It has been described that GK2 plays a crucial role in coordinating the arrangement of mitochondria during the formation of the mitochondrial sheath in mouse spermatozoa. Depletion or deficiencies in GK2 function have been observed to result in various consequential effects on sperm morphology and function [87,88]. Mechanistically, GK2 engages in multiple protein–protein interactions within the sperm cell. For instance, GK2 has been shown to interact with armadillo repeat-containing 12 (ARMC12), a protein implicated in mitochondrial elongation and coiling around the flagellum during spermatogenesis [89]. Additionally, GK2 binds to TBC1 domain family member 21 (TBC1D21), a spermatid-specific protein that contributes critically to mitochondrial sheath assembly in mammalian spermatogenesis [90]. Another significant interaction involves Phospholipase D family member 6 (PLD6), a mitochondrial surface enzyme responsible for the generation of phosphatidic acid (PA), a lipid signaling molecule involved in mitochondrial division and fusion. The interaction between GK2 and PLD6 facilitates the aggregation of PLD6 PA-dependent mitochondria within cells [91]. These findings establish GK2 as a key regulator of mitochondrial organization and dynamics in spermatozoa. Its interactions with critical mitochondrial and spermatogenesis-associated proteins highlight its essential role in the structural formation and functional integrity of the mitochondrial sheath, revealing GK2 to be a pivotal element of sperm maturation and male fertility.

The role of GK2 in the formation of the mitochondrial sheath seems to be vital. The absence of this enzyme manifests as significant defects in cellular differentiation occurring during spermatogenesis, resulting in the production of spermatozoa with aberrant morphology, particularly within the tail region. This structural anomaly renders the sperm incapable of normal motility, thereby compromising their ability to effectively fertilize the oocyte [87,88].

Given the binding of GK2 to mitochondria, an interaction with voltage-dependent anion channel (VDAC) proteins has been proposed [92], which serve as the principal pathways for ATP and ADP exchange across the outer mitochondrial membrane [93]. Notably, only VDAC2 and VDAC3 were described in spermatozoa, with VDAC2 being present in various regions, including the outer mitochondrial membrane, and VDAC3 restricted to the acrosome [94]. The activity and function of VDAC are regulated by its binding to various proteins (putatively including GK2) [92,95]. When this interaction is disrupted or impaired, the supply of ATP for extramitochondrial processes might be compromised [96], impacting ATP-dependent events such as sperm motility.

**Figure 3:**
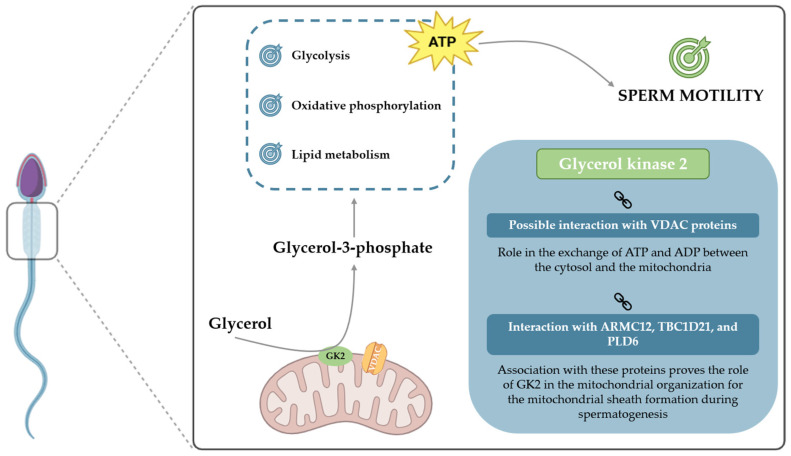
The dual role of glycerol kinase 2 (GK2) in sperm metabolism and structural organization. Located in the sperm midpiece, GK2 catalyzes the phosphorylation of glycerol into glycerol-3-phosphate (G3P). G3P is a crucial metabolic intermediate that fuels energy production through glycolysis, oxidative phosphorylation, and lipid metabolism, ultimately generating the ATP required for sperm motility. Beyond its metabolic role, GK2 is involved in key molecular interactions. Its putative association with VDAC proteins on the mitochondrial membrane may regulate the ATP/ADP exchange, while its interactions with ARMC12, TBC1D21, and PLD6 are critical for the structural organization of the mitochondrial sheath during spermatogenesis.

Moreover, in the context of a lipid-rich diet, the consequential increased availability of free fatty acids inhibits glycolysis and shifts metabolism towards lipogenesis and glyceroneogenesis [80,97,98]. This cascade of events leads to an increase in glycerol in various human tissues, initiating the process of fat accumulation. The heightened concentration of glycerol, as a result of these metabolic alterations, has the potential to adversely affect the BTB, leading to structural damage and anomalies in its functionality [99]. On the other hand, Siva and colleagues [71] observed an increase in the expression of GK2 in individuals with asthenozoospermia compared to those with normozoospermia. These results suggest that GK2 may play a compensatory role in response to high glycerol concentrations. This implies that GK2 could impact sperm motility beyond its catalytic properties, possibly acting as a link between metabolic pathways and the reproductive potential of males (Figure 3).

In addition to dietary factors, exposure to environmental toxicants can also disrupt glycerol homeostasis, as observed in certain species. For instance, studies in marine invertebrates have shown that exposure to heavy metals, such as cadmium, can lead to a significant increase in glycerol levels within the gonad [100]. While this may represent an adaptive response in those organisms, it highlights that glycerol metabolism is sensitive to environmental stressors. Given that precise glycerol concentrations are critical for maintaining the BTB in mammals [13,99], any environmentally induced variation could have significant pathological consequences, representing another mechanism through which toxicant exposure may impair male fertility.

**Table 1:** Overview of studies regarding the involvement of GK2 in different organisms and matrices.

Organism	Matrix	Results	Outcomes/Findings	Reference
**Mous** **e**	Sperm	• Abnormal localization of crescent-like mitochondria is shown in GK2-disrupted spermatids. • Disorganization of the mitochondrial sheath is reported in GK2-disrupted mice.	GK2 is essential for the arrangement of crescent-like mitochondria to form the mitochondrial sheath during spermatogenesis.	Shimada et al. [88]
**Mouse**	Sperm	• ARMC12 interacts with GK2 (and other mitochondrial proteins, such as VDAC), which is required for mitochondrial sheath formation. • Absence of ARMC12 causes abnormal mitochondrial coiling around the flagellum, resulting in reduced motility and infertility.	GK2 interaction with ARMC12 shows that it is involved in mitochondrial dynamics to form the mitochondrial sheath.	Shimada et al. [89]
**Mouse**	Sperm	• GK2 binds to TBC1D21, a protein that contributes to the mitochondrial sheath assembly during spermatogenesis. • TBC1D21 KO-mice showed mitochondrial sheath malformation.	Association with TBC1D21 proves the involvement of GK2 in the formation of the mitochondrial sheath.	Chen et al. [90]
**Bovine**	Testis	• GK2 interacts with PLD6, a phospholipase responsible for the production of PA in the mitochondrial surface (playing a role in the regulation of mitochondrial division and fusion). • PLD6 also plays a role in the production of piRNA, which is directly involved in the regulation of male germ cell development.	GK2 association with PLD6 suggests that, as PLD6 has a pivotal role in the development of male germ cells, this enzyme may also be involved in this process.	Yang et al. [91]
**Mouse**	Sperm	• GK2 seems to interact with Kastor and Polluks, two sperm-specific polypeptides that are directly connected with VDAC proteins. • VDAC3-deficient mice show reduced sperm motility and consequent infertility.	Presents a putative interaction between GK2 and VDAC proteins and a consequent role of GK2 in ATP exchanges in the mitochondrial membrane necessary to sustain motility.	Mise et al. [92]
**Human**	Sperm	• Proteomic analysis shows that a group of proteins related to “energy and metabolism”, including GK2, is increased in asthenozoospermic individuals.	Increased GK2 expression in asthenozoospermic individuals may be caused by incomplete maturation, causing more redundant cytoplasm and, therefore, more cytosolic proteins.	Siva et al. [71]

### 5.2. Methodologies for Evaluating GK2 as a Fertility Biomarker

Given the potential interaction between GK2 and VDAC, which may directly involve GK2 in ATP exchange between the cytosol and mitochondria, evaluating both the presence and enzymatic activity of GK2 becomes essential. It can be hypothesized that higher GK2 activity in the sperm midpiece enhances ATP production and transport, thereby sustaining sperm motility. As a result, increased motility would be expected due to the greater availability of energy, facilitating the selection of the most competent spermatozoa for use in ARTs. Consequently, assessing GK2 expression and activity could represent a pivotal step toward optimizing sperm selection processes in ARTs.

Various approaches exist for evaluating these parameters. In the context of protein expression, multiple methodologies can be employed. Western blotting is a commonly used technique that enables the detection of specific proteins in sperm samples based on their molecular weight and allows for their quantification [101]. Microscopy-based analyses, such as immunofluorescence, can further confirm the presence and subcellular localization of the protein of interest [101]. Additionally, mass spectrometry provides a powerful tool for sperm proteome profiling, allowing the identification of thousands of proteins and the comparison of protein expression between fertile and infertile sperm samples [71,102]. Regarding protein activity, enzymatic activity assays remain the primary approach for directly quantifying enzyme activity by measuring the conversion of substrate to product. Currently, only a limited number of methods are available for assessing GK2-specific activity, although techniques for general glycerol kinase activity quantification have been established [103].

## 6. Conclusions and Future Prospects

Despite the still unclear role of GK2 in sperm motility, this enzyme emerges as a promising biomarker for sperm selection in (ARTs. Its involvement in sperm bioenergetics suggests a potential impact on fertility, making it a subject of great interest in reproductive medicine.

GK2 plays a crucial role in the G3P shuttle, catalyzing the phosphorylation of glycerol into G3P—an essential metabolite in various biochemical pathways within spermatozoa. This enzymatic activity contributes significantly to ATP production, which is fundamental for supporting multiple sperm functions, particularly motility. Given that ATP is the primary energy source for flagellar movement, the regulation of GK2 activity may have direct implications for sperm performance and fertilization potential.

Certain limitations should be considered when interpreting the findings of this review. This work is a narrative, rather than a systematic review, and thus may be subject to selection bias. Furthermore, as highlighted, a significant portion of the evidence regarding GK2’s fundamental role in spermatogenesis is derived from animal models. While these studies are invaluable, the direct extrapolation of their findings to human sperm physiology requires caution. Consequently, the proposition of GK2 as a clinical biomarker for male infertility, while promising, remains a prospective goal that necessitates further validation through dedicated human studies.

The use of the Proximity-Ligation Assay (PLA) represents a promising approach to further elucidate the specific involvement of GK2 in sperm motility. This technique could facilitate an investigation into a potential interaction between GK2 and VDAC proteins, which are key regulators of mitochondrial function. A confirmed association between GK2 and VDAC proteins would strongly suggest that GK2 plays a role in mitochondrial ATP production and energy distribution within spermatozoa. This would further support the hypothesis that GK2 contributes to motility by ensuring an adequate supply of ATP for efficient flagellar movement.

Confirmation of this interaction would represent a highly promising finding, positioning GK2 as a potential biomarker of significant value for sperm selection in ART procedures. Sperm samples exhibiting elevated GK2 activity could potentially be prioritized, as they would likely display enhanced motility, thereby improving the probability of successful fertilization. By integrating GK2 assessment into sperm selection criteria, ARTs could benefit from more refined and effective strategies for optimizing reproductive outcomes.

To translate the promising findings discussed herein into clinical practice, future research should be directed toward clear, sequential goals. The primary objective must definitively confirm the putative GK2-VDAC interaction in human spermatozoa using techniques such as the PLA. Following this, the next step will be to elucidate the direct functional impact of this protein complex on sperm bioenergetics and motility. Ultimately, achieving these goals will pave the way for the development of a validated GK2-based biomarker assay for optimizing sperm selection in ARTs, thereby improving outcomes for male infertility.

## Data Availability

Not applicable.

## Abbreviations

The following abbreviations are used in this manuscript:

ADP	Adenosine diphosphate
AKAPs	A-kinase anchor proteins
AQP7	Aquaglyceroporin-7
ARTs	Assisted reproductive technologies
ARMC12	Armadillo repeat-containing 12
ATP BTB	Adenosine triphosphate Blood–testis barrier
cAMP	Cyclic adenosine monophosphatase
CDC	Centers for Disease Control
CFTR	Cystic fibrosis transmembrane conductance regulator
DC	Distal centriole
DHAP	Dihydroxyacetone phosphate
ETC	Electron transport chain
FAD	Flavin adenine dinucleotide
FADH2	Flavin adenine dinucleotide
FFAs	Free fatty acids
FSs	Fibrous sheets
FSH	Follicle-stimulating hormone
G3P	Glycerol-3-phosphate
GAPDH	Glyceraldehyde-3-phosphate dehydrogenase
GK	Glycerol kinase
GK1	Glycerol kinase 1
GK2	Glycerol kinase 2
GnRH	Gonadotropin-releasing hormone
GSK3	Glycogen synthase kinase 3
ICSI	Intracytoplasmic sperm injection
IVF	*In vitro* fertilization
LH	Luteinizing hormone
LRP6	Lipoprotein receptor-related protein 6 receptor
NAD+	Nicotinamide adenine dinucleotide
NADH	Reduce nicotinamide adenine dinucleotide
PA	Phosphatidic acid
PC	Proximal centriole
PDPK1	3-phosphoinositide-dependent protein kinase 1
PGT	Preimplantation genetic testing
PIP2	Phosphatidylinositol 4,5-bisphosphate
PIP3	Phosphatidylinositol 3,4,5-trisphosphate
PLA	Proximity-ligation assay
PLD6	Phospholipase D family member 6
PPME1	Protein phosphatase methylesterase 1
PPP	Pentose phosphate pathway
PPP1	Phosphoprotein phosphatase 1
PPP1R2	PPP1 regulatory subunit 2
PPP1R2P3	PPP1 regulatory subunit 2 isoform
PPP2A	Phosphoprotein phosphatase 2A
PPP2CA	Phosphoprotein phosphatase 2A isoform alpha
PRKA	Protein kinase A
ROS	Reactive oxygen species
sAC	Soluble adenylyl cyclase
TBC1D21	TBC1 domain family member 21
TCA	Tricarboxylic acid
VDAC	Voltage-dependent anion channel
WHO	World Health Organization

## References

[1] Clavijo R.I., Hsiao W. (2018). Update on male reproductive endocrinology. Transl. Androl. Urol..

[2] Huang B., Wang Z., Kong Y., Jin M., Ma L. (2023). Global, regional and national burden of male infertility in 204 countries and territories between 1990 and 2019: An analysis of global burden of disease study. BMC Public Health.

[3] Agarwal A., Baskaran S., Parekh N., Cho C.L., Henkel R., Vij S., Arafa M., Panner Selvam M.K., Shah R. (2021). Male infertility. Lancet.

[4] Ghuman N., Ramalingam M. (2018). Male infertility. Obstet. Gynaecol. Reprod. Med..

[5] Kose S.I. (2023). Imaging in Male Infertility. Curr. Probl. Diagn. Radiol..

[6] Merchant R., Gandhi G., Allahbadia G.N. (2011). In vitro fertilization/intracytoplasmic sperm injection for male infertility. Indian J. Urol..

[7] Begum M. (2010). Assisted Reproductive Technology: Techniques and Limitations. J. Bangladesh Coll. Physicians Surg..

[8] World Health Organization (2021). WHO Laboratory Manual for the Examination and Processing of Human Semen.

[9] Lobo N., Satchi M. (2019). The diagnosis and management of men with low sperm motility. Trends Urol. Men’s Health.

[10] Moreira R.J., Oliveira P.F., Spadella M.A., Ferreira R., Alves M.G. (2025). Do Lifestyle Interventions Mitigate the Oxidative Damage and Inflammation Induced by Obesity in the Testis?. Antioxidants.

[11] Esteves S.C. (2022). Evolution of the World Health Organization semen analysis manual: Where are we?. Nat. Rev. Urol..

[12] Perrone P., Lettieri G., Marinaro C., Longo V., Capone S., Forleo A., Pappalardo S., Montano L., Piscopo M. (2022). Molecular Alterations and Severe Abnormalities in Spermatozoa of Young Men Living in the “Valley of Sacco River” (Latium, Italy): A Preliminary Study. Int. J. Environ. Res. Public Health.

[13] Crisóstomo L., Alves M.G., Calamita G., Sousa M., Oliveira P.F. (2017). Glycerol and testicular activity: The good, the bad and the ugly. Mol. Hum. Reprod..

[14] Sargent C.A., Young C., Marsh S., Ferguson-Smith M.A., Affara N.A. (1994). The glycerol kinase gene family: Structure of the Xp gene, and related intronless retroposons. Hum. Mol. Genet..

[15] Peroni O., Large V., Beylot M. (1995). Measuring gluconeogenesis with [2–13C]glycerol and mass isotopomer distribution analysis of glucose. Am. J. Physiol..

[16] Assisted Reproductive Technology (ART) Centers for Disease Control and Prevention (CDC). https://www.cdc.gov/art/about/index.html.

[17] Liberman R.F., Getz K.D., Heinke D., Luke B., Stern J.E., Declercq E.R., Chen X., Lin A.E., Anderka M. (2017). Assisted Reproductive Technology and Birth Defects: Effects of Subfertility and Multiple Births. Birth Defects Res..

[18] Davidovitch M., Chodick G., Shalev V., Eisenberg V.H., Dan U., Reichenberg A., Sandin S., Levine S.Z. (2018). Infertility treatments during pregnancy and the risk of autism spectrum disorder in the offspring. Prog. Neuropsychopharmacol. Biol. Psychiatry.

[19] Hattori H., Hiura H., Kitamura A., Miyauchi N., Kobayashi N., Takahashi S., Okae H., Kyono K., Kagami M., Ogata T. (2019). Association of four imprinting disorders and ART. Clin. Epigenetics.

[20] Njagi P., Groot W., Arsenijevic J., Dyer S., Mburu G., Kiarie J. (2023). Financial costs of assisted reproductive technology for patients in low- and middle-income countries: A systematic review. Hum. Reprod. Open.

[21] Sallam H.N., Sallam N.H. (2016). Religious aspects of assisted reproduction. Facts Views Vis. ObGyn.

[22] Grin L., Girsh E., Harlev A. (2021). Male fertility preservation-Methods, indications and challenges. Andrologia.

[23] Albamonte M.I., Vitullo A.D. (2023). Preservation of fertility in female and male prepubertal patients diagnosed with cancer. J. Assist. Reprod. Genet..

[24] Fesahat F., Montazeri F., Hoseini S.M. (2020). Preimplantation genetic testing in assisted reproduction technology. J. Gynecol. Obstet. Hum. Reprod..

[25] Greco E., Litwicka K., Minasi M.G., Cursio E., Greco P.F., Barillari P. (2020). Preimplantation Genetic Testing: Where We Are Today. Int. J. Mol. Sci..

[26] Raja N.S., Russell C.B., Moravek M.B. (2022). Assisted reproductive technology: Considerations for the nonheterosexual population and single parents. Fertil. Steril..

[27] Sharma R., Agarwal A. (2011). Spermatogenesis: An overview. Sperm Chromatin: Biological and Clinical Applications in Male Infertility and Assisted Reproduction.

[28] de Kretser D.M., Loveland K.L., Meinhardt A., Simorangkir D., Wreford N. (1998). Spermatogenesis. Hum. Reprod..

[29] Hermo L., Lalli M., Clermont Y. (1977). Arrangement of connective tissue components in the walls of seminiferous tubules of man and monkey. Am. J. Anat..

[30] Berezney R., Coffey D.S. (1977). Nuclear matrix. Isolation and characterization of a framework structure from rat liver nuclei. J. Cell Biol..

[31] Neto F.T.L., Bach P.V., Najari B.B., Li P.S., Goldstein M. (2016). Spermatogenesis in humans and its affecting factors. Semin. Cell Dev. Biol..

[32] Freitas M.J., Vijayaraghavan S., Fardilha M. (2017). Signaling mechanisms in mammalian sperm motility. Biol. Reprod..

[33] Barratt C.L., Aitken R.J., Björndahl L., Carrell D.T., de Boer P., Kvist U., Lewis S.E., Perreault S.D., Perry M.J., Ramos L. (2010). Sperm DNA: Organization, protection and vulnerability: From basic science to clinical applications--a position report. Hum. Reprod..

[34] Piomboni P., Focarelli R., Stendardi A., Ferramosca A., Zara V. (2012). The role of mitochondria in energy production for human sperm motility. Int. J. Androl..

[35] Lehti M.S., Sironen A. (2017). Formation and function of sperm tail structures in association with sperm motility defects. Biol. Reprod..

[36] Eddy E.M. (2007). The scaffold role of the fibrous sheath. Soc. Reprod. Fertil. Suppl..

[37] Amaro A., Bernardo C. (2015). Reprodução Humana Masculina—Princípios Fundamentais.

[38] Korrodi-Gregório L., Ferreira M., Vintém A.P., Wu W., Muller T., Marcus K., Vijayaraghavan S., Brautigan D.L., da Cruz E.S.O.A., Fardilha M. (2013). Identification and characterization of two distinct PPP1R2 isoforms in human spermatozoa. BMC Cell Biol..

[39] Vijayaraghavan S., Stephens D.T., Trautman K., Smith G.D., Khatra B., da Cruz e Silva E.F., Greengard P. (1996). Sperm motility development in the epididymis is associated with decreased glycogen synthase kinase-3 and protein phosphatase 1 activity. Biol. Reprod..

[40] Koch S., Acebron S.P., Herbst J., Hatiboglu G., Niehrs C. (2015). Post-transcriptional Wnt Signaling Governs Epididymal Sperm Maturation. Cell.

[41] Wang Q.M., Park I.K., Fiol C.J., Roach P.J., DePaoli-Roach A.A. (1994). Isoform differences in substrate recognition by glycogen synthase kinases 3 alpha and 3 beta in the phosphorylation of phosphatase inhibitor 2. Biochemistry.

[42] Dudiki T., Kadunganattil S., Ferrara J.K., Kline D.W., Vijayaraghavan S. (2015). Changes in Carboxy Methylation and Tyrosine Phosphorylation of Protein Phosphatase PP2A Are Associated with Epididymal Sperm Maturation and Motility. PLoS ONE.

[43] Mortimer D., Mortimer S. (2005). Laboratory Investigation of the Infertile Male.

[44] Chemes H., Olmedo S.B., Carrere C., Oses R., Carizza C., Leisner M., Blaquier J. (1998). Ultrastructural pathology of the sperm flagellum: Association between flagellar pathology and fertility prognosis in severely asthenozoospermic men. Hum. Reprod..

[45] Damsgaard J., Joensen U.N., Carlsen E., Erenpreiss J., Blomberg Jensen M., Matulevicius V., Zilaitiene B., Olesen I.A., Perheentupa A., Punab M. (2016). Varicocele Is Associated with Impaired Semen Quality and Reproductive Hormone Levels: A Study of 7035 Healthy Young Men from Six European Countries. Eur. Urol..

[46] Diemer T., Huwe P., Ludwig M., Hauck E., Weidner W. (2003). Urogenital infection and sperm motility. Andrologia.

[47] Pattinson H.A., Mortimer D. (1987). Prevalence of sperm surface antibodies in the male partners of infertile couples as determined by immunobead screening. Fertil. Steril..

[48] Aitken R.J., Buckingham D.W., Brindle J., Gomez E., Baker H.W., Irvine D.S. (1995). Analysis of sperm movement in relation to the oxidative stress created by leukocytes in washed sperm preparations and seminal plasma. Hum. Reprod..

[49] Lettieri G., D’Agostino G., Mele E., Cardito C., Esposito R., Cimmino A., Giarra A., Trifuoggi M., Raimondo S., Notari T. (2020). Discovery of the Involvement in DNA Oxidative Damage of Human Sperm Nuclear Basic Proteins of Healthy Young Men Living in Polluted Areas. Int. J. Mol. Sci..

[50] Nunzio A.D., Giarra A., Toscanesi M., Amoresano A., Piscopo M., Ceretti E., Zani C., Lorenzetti S., Trifuoggi M., Montano L. (2022). Comparison between Macro and Trace Element Concentrations in Human Semen and Blood Serum in Highly Polluted Areas in Italy. Int. J. Environ. Res. Public Health.

[51] Rotimi D.E., Singh S.K. (2024). Implications of lifestyle factors on male reproductive health. JBRA Assist. Reprod..

[52] Turner R.M. (2003). Tales from the tail: What do we really know about sperm motility?. J. Androl..

[53] du Plessis S.S., Agarwal A., Mohanty G., van der Linde M. (2015). Oxidative phosphorylation versus glycolysis: What fuel do spermatozoa use?. Asian J. Androl..

[54] Hereng T.H., Elgstøen K.B., Cederkvist F.H., Eide L., Jahnsen T., Skålhegg B.S., Rosendal K.R. (2011). Exogenous pyruvate accelerates glycolysis and promotes capacitation in human spermatozoa. Hum. Reprod..

[55] Mukai C., Okuno M. (2004). Glycolysis plays a major role for adenosine triphosphate supplementation in mouse sperm flagellar movement. Biol. Reprod..

[56] Williams A.C., Ford W.C. (2001). The role of glucose in supporting motility and capacitation in human spermatozoa. J. Androl..

[57] Ferramosca A., Provenzano S.P., Coppola L., Zara V. (2012). Mitochondrial respiratory efficiency is positively correlated with human sperm motility. Urology.

[58] Paoli D., Gallo M., Rizzo F., Baldi E., Francavilla S., Lenzi A., Lombardo F., Gandini L. (2011). Mitochondrial membrane potential profile and its correlation with increasing sperm motility. Fertil. Steril..

[59] Ford W.C. (2006). Glycolysis and sperm motility: Does a spoonful of sugar help the flagellum go round?. Hum. Reprod. Update.

[60] Dias T.R., Alves M.G., Silva B.M., Oliveira P.F. (2014). Sperm glucose transport and metabolism in diabetic individuals. Mol. Cell. Endocrinol..

[61] Amaral A. (2022). Energy metabolism in mammalian sperm motility. WIREs Mech. Dis..

[62] Amaral A., Castillo J., Estanyol J.M., Ballescà J.L., Ramalho-Santos J., Oliva R. (2013). Human sperm tail proteome suggests new endogenous metabolic pathways. Mol. Cell. Proteom..

[63] Amaral A., Paiva C., Baptista M., Sousa A.P., Ramalho-Santos J. (2011). Exogenous glucose improves long-standing human sperm motility, viability, and mitochondrial function. Fertil. Steril..

[64] Silva R., Carrageta D.F., Alves M.G., Oliveira P.F. (2022). Testicular Glycogen Metabolism: An Overlooked Source of Energy for Spermatogenesis?. BioChem.

[65] Miki K. (2007). Energy metabolism and sperm function. Soc. Reprod. Fertil. Suppl..

[66] Chaves V.E., Frasson D., Garófalo M.A., Navegantes L.C., Migliorini R.H., Kettelhut I.C. (2012). Increased glyceride-glycerol synthesis in liver and brown adipose tissue of rat: In-vivo contribution of glycolysis and glyceroneogenesis. Lipids.

[67] Christoffersen C., Bollano E., Lindegaard M.L., Bartels E.D., Goetze J.P., Andersen C.B., Nielsen L.B. (2003). Cardiac lipid accumulation associated with diastolic dysfunction in obese mice. Endocrinology.

[68] Jin E.S., Sherry A.D., Malloy C.R. (2014). Interaction between the pentose phosphate pathway and gluconeogenesis from glycerol in the liver. J. Biol. Chem..

[69] Mugabo Y., Zhao S., Seifried A., Gezzar S., Al-Mass A., Zhang D., Lamontagne J., Attane C., Poursharifi P., Iglesias J. (2016). Identification of a mammalian glycerol-3-phosphate phosphatase: Role in metabolism and signaling in pancreatic β-cells and hepatocytes. Proc. Natl. Acad. Sci. USA.

[70] Ratner P.L., Fisher M., Burkart D., Cook J.R., Kozak L.P. (1981). The role of mRNA levels and cellular localization in controlling sn-glycerol-3-phosphate dehydrogenase expression in tissues of the mouse. J. Biol. Chem..

[71] Siva A.B., Kameshwari D.B., Singh V., Pavani K., Sundaram C.S., Rangaraj N., Deenadayal M., Shivaji S. (2010). Proteomics-based study on asthenozoospermia: Differential expression of proteasome alpha complex. Mol. Hum. Reprod..

[72] Kosuga M., Henderson-MacLennan N.K., Zhang Y.H., Huang B.L., Dipple K.M., McCabe E.R. (2011). Glycerol homeostasis and metabolism in glycerol kinase carrier mice. Mol. Genet. Metab..

[73] Wikiera B., Jakubiak A., Zimowski J., Noczyńska A., Smigiel R. (2012). Complex glycerol kinase deficiency—X-linked contiguous gene syndrome involving congenital adrenal hypoplasia, glycerol kinase deficiency, muscular Duchenne dystrophy and intellectual disability (IL1RAPL gene deletion). Pediatr. Endocrinol. Diabetes Metab..

[74] Green D.E. (1936). Alpha-Glycerophosphate dehydrogenase. Biochem. J..

[75] Houstĕk J., Drahota Z. (1975). The regulation of glycerol 3-phosphate oxidase of rate brownadipose tissue mitochondria by long-chain free fatty acids. Mol. Cell. Biochem..

[76] Wu J.W., Yang H., Wang S.P., Soni K.G., Brunel-Guitton C., Mitchell G.A. (2015). Inborn errors of cytoplasmic triglyceride metabolism. J. Inherit. Metab. Dis..

[77] Hibuse T., Maeda N., Nakatsuji H., Tochino Y., Fujita K., Kihara S., Funahashi T., Shimomura I. (2009). The heart requires glycerol as an energy substrate through aquaporin 7, a glycerol facilitator. Cardiovasc. Res..

[78] Mohammad M.A., Maningat P., Sunehag A.L., Haymond M.W. (2015). Precursors of hexoneogenesis within the human mammary gland. Am. J. Physiol.-Endocrinol. Metab..

[79] Nye C.K., Hanson R.W., Kalhan S.C. (2008). Glyceroneogenesis is the dominant pathway for triglyceride glycerol synthesis in vivo in the rat. J. Biol. Chem..

[80] Bukowiecki L.J., Lindberg O. (1974). Control of sn-glycerol 3-phosphate oxidation in brown adipose tissue mitochondria by calcium and acyl-CoA. Biochim. Biophys. Acta.

[81] Garrib A., McMurray W.C. (1986). Purification and characterization of glycerol-3-phosphate dehydrogenase (flavin-linked) from rat liver mitochondria. J. Biol. Chem..

[82] Mráček T., Holzerová E., Drahota Z., Kovářová N., Vrbacký M., Ješina P., Houštěk J. (2014). ROS generation and multiple forms of mammalian mitochondrial glycerol-3-phosphate dehydrogenase. Biochim. Biophys. Acta.

[83] Mráček T., Drahota Z., Houštěk J. (2013). The function and the role of the mitochondrial glycerol-3-phosphate dehydrogenase in mammalian tissues. Biochim. Biophys. Acta.

[84] Ribeiro J.C., Bernardino R.L., Gonçalves A., Barros A., Calamita G., Alves M.G., Oliveira P.F. (2023). Aquaporin-7-Mediated Glycerol Permeability Is Linked to Human Sperm Motility in Asthenozoospermia and during Sperm Capacitation. Cells.

[85] Ribeiro J.C., Bernardino R.L., Carrageta D.F., Soveral G., Calamita G., Alves M.G., Oliveira P.F. (2022). CFTR modulates aquaporin-mediated glycerol permeability in mouse Sertoli cells. Cell. Mol. Life Sci..

[86] Kota V., Dhople V.M., Shivaji S. (2009). Tyrosine phosphoproteome of hamster spermatozoa: Role of glycerol-3-phosphate dehydrogenase 2 in sperm capacitation. Proteomics.

[87] Chen Y., Liang P., Huang Y., Li M., Zhang X., Ding C., Feng J., Zhang Z., Zhang X., Gao Y. (2017). Glycerol kinase-like proteins cooperate with Pld6 in regulating sperm mitochondrial sheath formation and male fertility. Cell Discov..

[88] Shimada K., Kato H., Miyata H., Ikawa M. (2019). Glycerol kinase 2 is essential for proper arrangement of crescent-like mitochondria to form the mitochondrial sheath during mouse spermatogenesis. J. Reprod. Dev..

[89] Shimada K., Park S., Miyata H., Yu Z., Morohoshi A., Oura S., Matzuk M.M., Ikawa M. (2021). ARMC12 regulates spatiotemporal mitochondrial dynamics during spermiogenesis and is required for male fertility. Proc. Natl. Acad. Sci. USA.

[90] Chen Y., Chen X., Zhang H., Sha Y., Meng R., Shao T., Yang X., Jin P., Zhuang Y., Min W. (2022). TBC1D21 is an essential factor for sperm mitochondrial sheath assembly and male fertility. Biol. Reprod..

[91] Yang R., Zhang B., Zhu W., Zhu C., Chen L., Zhao Y., Wang Y., Zhang Y., Riaz A., Tang B. (2023). Expression of Phospholipase D Family Member 6 in Bovine Testes and Its Molecular Characteristics. Int. J. Mol. Sci..

[92] Mise S., Matsumoto A., Shimada K., Hosaka T., Takahashi M., Ichihara K., Shimizu H., Shiraishi C., Saito D., Suyama M. (2022). Kastor and Polluks polypeptides encoded by a single gene locus cooperatively regulate VDAC and spermatogenesis. Nat. Commun..

[93] Fiek C., Benz R., Roos N., Brdiczka D. (1982). Evidence for identity between the hexokinase-binding protein and the mitochondrial porin in the outer membrane of rat liver mitochondria. Biochim. Biophys. Acta (BBA)—Biomembr..

[94] Kwon W.-S., Park Y.-J., Mohamed E.-S.A., Pang M.-G. (2013). Voltage-dependent anion channels are a key factor of male fertility. Fertil. Steril..

[95] Liu B., Zhang W., Wang Z. (2010). Voltage-dependent anion channel in mammalian spermatozoa. Biochem. Biophys. Res. Commun..

[96] McCabe E.R. (1983). Human glycerol kinase deficiency: An inborn error of compartmental metabolism. Biochem. Med..

[97] Patsouris D., Mandard S., Voshol P.J., Escher P., Tan N.S., Havekes L.M., Koenig W., März W., Tafuri S., Wahli W. (2004). PPARalpha governs glycerol metabolism. J. Clin. Investig..

[98] Seidler N.W. (2013). GAPDH and intermediary metabolism. Adv. Exp. Med. Biol..

[99] Wiebe J.P., Kowalik A., Gallardi R.L., Egeler O., Clubb B.H. (2000). Glycerol disrupts tight junction-associated actin microfilaments, occludin, and microtubules in Sertoli cells. J. Androl..

[100] Lettieri G., Marinaro C., Brogna C., Montano L., Lombardi M., Trotta A., Troisi J., Piscopo M. (2023). A Metabolomic Analysis to Assess the Responses of the Male Gonads of Mytilus galloprovincialis after Heavy Metal Exposure. Metabolites.

[101] Bucci D., Spinaci M., Galeati G., Tamanini C. (2020). Different approaches for assessing sperm function. Anim. Reprod..

[102] Cao X., Cui Y., Zhang X., Lou J., Zhou J., Bei H., Wei R. (2018). Proteomic profile of human spermatozoa in healthy and asthenozoospermic individuals. Reprod. Biol. Endocrinol..

[103] Glycerol Kinase—Assay. https://www.worthington-biochem.com/products/glycerol-kinase/assay.

